# Prolonged oral vancomycin for secondary prophylaxis of relapsing *Clostridium difficile* infection

**DOI:** 10.1186/s12879-019-3676-1

**Published:** 2019-01-14

**Authors:** Kevin Zhang, Patricia Beckett, Salaheddin Abouanaser, Vida Stankus, Christine Lee, Marek Smieja

**Affiliations:** 1 Bachelor of Health Sciences Program, McMaster University, Hamilton, ON Canada; 2 St. Joseph’s Healthcare Hamilton, L424-St., 50 Charlton Ave. E, Hamilton, ON L8N 4A6 Canada; 3 Department of Pathology and Molecular Medicine, McMaster University, Hamilton, ON Canada; 4 Royal Jubilee Hospital, Victoria, BC Canada; 5 Michael G. DeGroote Institute of Infectious Diseases Research, McMaster University, Hamilton, ON Canada

**Keywords:** Clostridium difficile, Relapse-prevention, Case series, Prophylaxis, Vancomycin

## Abstract

**Background:**

*Clostridium difficile* infection (CDI) is an important cause of diarrhea and continues to be a major burden within healthcare institutions and in the community. For a small subset of patients with frequently relapsing CDI who do not have access to fecal microbiota transplantation (FMT), or fail FMT, there are no clear treatment recommendations. We review our experience with prolonged oral vancomycin for secondary prophylaxis of relapsing CDI.

**Methods:**

We performed a retrospective chart review of cases from the *C. difficile* consultation service at our institution since 2013. The service had three primary physicians providing consultations and performing over 1000 FMTs over the five-year period. Patients with relapsing CDI who were not candidates for FMT, refused, or relapsed after FMT were treated with vancomycin, followed by long-term oral vancomycin at a dose of 125 mg once daily.

**Results:**

Twenty patients received at least 8 weeks of once-daily oral vancomycin for prophylaxis of relapsing CDI. Patients had a median age of 80 years, and experienced a median of four episodes of CDI prior to long-term vancomycin. Most were female and 75% had received FMT. Only a single case of *C. difficile* relapse occurred while on long-term vancomycin during 200 patient-months of follow-up. Amongst those who stopped long-term vancomycin, 31% relapsed within 6 weeks. No adverse events were observed.

**Conclusions:**

For elderly patients with frequently relapsing *C. difficile*, prolonged vancomycin once daily at a dose of 125 mg orally was effective in preventing further relapse. Vancomycin secondary prophylaxis may be considered in patients who have failed FMT, or in cases where FMT is not available.

## Background

*Clostridium difficile* infection (CDI) is an important cause of diarrhea and continues to be a major burden within healthcare institutions and in the community [1]. Recurrent CDI presents a significant clinical challenge with limited treatment options and is, moreover, associated with an elevated risk for future recurrence and severe disease [1]. In a large randomized trial conducted at our institution, both fresh and frozen fecal microbiota transplantation (FMT) were demonstrated to be effective treatment options for patients with multiple relapses or refractory CDI [2]. However, despite excellent access to FMT for our patients, up to 20% will fail or relapse and other treatment options are needed. Furthermore, in an electronic survey of over 600 physicians in 2012, only 24% had access to FMT in their institution, citing issues with logistics and complexities associated with donor screening [3]. In this case series, we summarize our experience with long-term vancomycin for CDI prophylaxis amongst patients with multiple episodes who failed FMT, or were not candidates for FMT.

St. Joseph’s Healthcare Hamilton is a teaching hospital in Hamilton, Ontario, Canada with a dedicated *Clostridium difficile* consultation service for inpatients and referred outpatients. The consultation team treats relapsing and severe CDI and may offer FMT by rectal enema to patients with multiple CDI episodes or refractory disease. In a small subset of patients, around 3% of referrals, who were not candidates for FMT or failed multiple FMT enemas, long-term vancomycin to suppress symptoms and prevent relapse was prescribed. We conducted a retrospective case-series of 20 consecutive cases from January 2013 to December 2017 to summarize our experience with long-term vancomycin for secondary prophylaxis of relapsing CDI. Inclusion criteria were defined as patients with three or more CDI episodes who were prescribed at least 8 weeks of oral vancomycin, at a dosage of 125 mg once daily to prevent CDI relapse, and who were not taking concomitant antibiotics for other infections. Vancomycin prophylaxis with concurrent antibiotics has been previously studied, using 125–250 mg vancomycin two to four times daily, and was effective against CDI recurrence in that setting [4, 5]. To our knowledge, however, there are no studies investigating the use of long-term vancomycin for greater than 8 weeks as secondary prophylaxis to prevent CDI relapse in those who are not taking concomitant antibiotics.

## Methods

We studied 20 consecutive patients, over the period of January 2013 to December 2017, who were prescribed 125 mg vancomycin orally once daily for at least 8 weeks; their demographics, prior CDI treatment, and comorbidities are summarized in the Table 1. Patients were identified from databases maintained by infection control and review of paperwork required for access to government funding. The median age was 80 years (range, 53–92 years) and 7 (35%) of the 20 patients were male. A CDI episode was characterised clinically as 3 or more type 5–7 diarrheal stools on the Bristol stool scale, together with a positive test for *Clostridium difficile* toxin gene using a laboratory-developed molecular test. Patients experienced a median of 4 CDI episodes (range, 2–11). Prior to long-term vancomycin, 18 (90%) received a median of 2 courses of oral metronidazole and 20 (100%) received a median of 3 courses of oral vancomycin. Following a 14-day treatment course of vancomycin, patients were tapered over 2 weeks to prolonged oral vancomycin at a dose of 125 mg once daily. Of the 15 patients who received a median of 3 FMTs by rectal enema (range, 1–12), 14 (93%) did not demonstrate a clinical response.

**Table 1:** Description of Patients on Once Daily Vancomycin Prophylaxis (*n* = 20)

Demographics	
Age, median (min, max)	80 years (53, 92)
Male	7 (35%)
*C. difficile* Infection (CDI) Episodes, median (min, max)	4 (2, 11)
Previous CDI Treatment	
Metronidazole	18 (90%)
Courses of Metronidazole, median (min, max)	2 (1, 5)
Vancomycin	20 (100%)
Courses of Vancomycin, median (min, max)	3 (1, 11)
Fidaxomicin	4 (20%)
Fecal Microbiota Transplantation (FMT)	15 (75%)
Number of FMTs, median (min, max)	3 (1, 12)
Comorbidities	
Cardiac	13 (65%)
Chronic Obstructive Pulmonary Disease	8 (40%)
Chronic Kidney Disease	7 (35%)
Diabetes Type 2	5 (25%)
Gastroesophageal Reflux Disease	7 (35%)

## Results

**Fig. 1:**
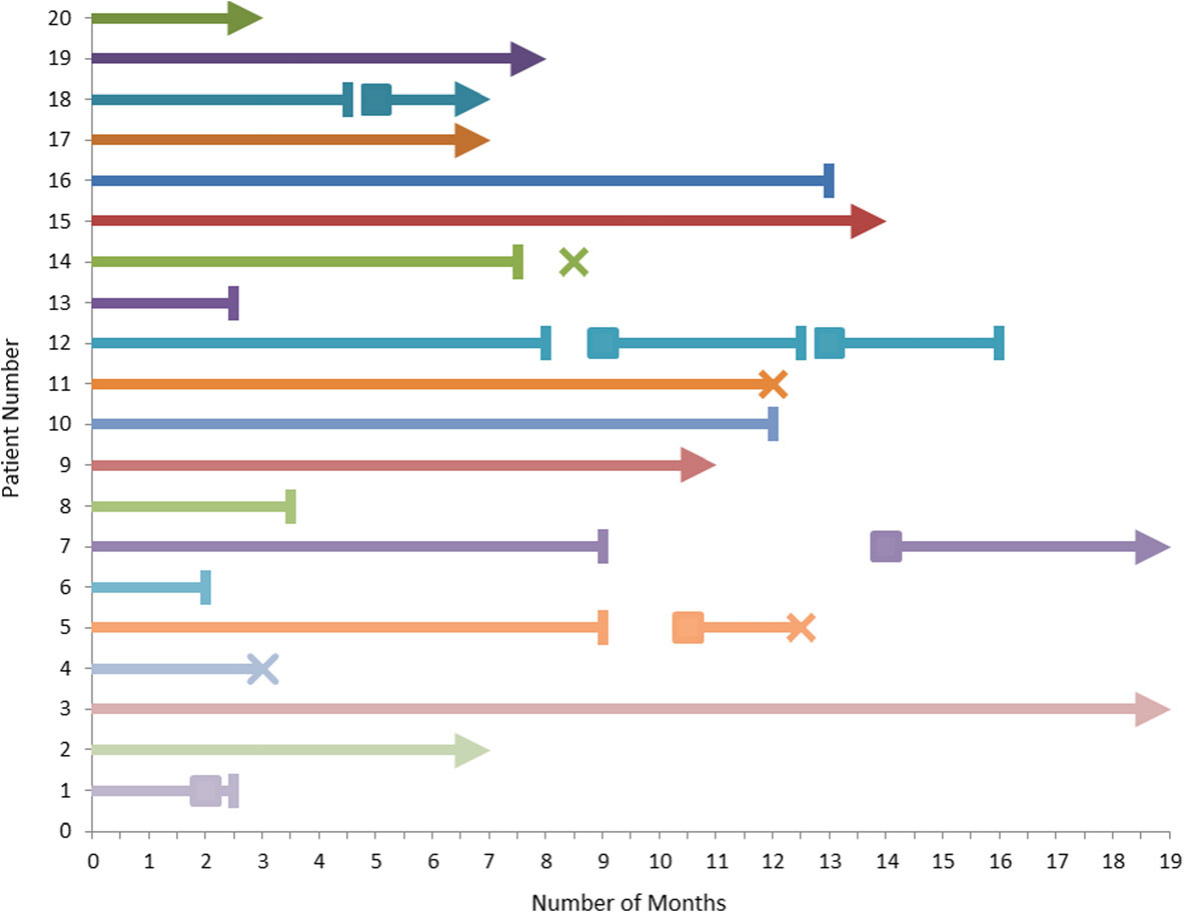
Effect of prolonged vancomycin 125 mg daily as secondary prophylaxis of relapsing *Clostridium difficile* infection. Legend:  *C. difficile* episode;  Death; Completed Vancomycin Prophylaxis; Ongoing Vancomycin Prophylaxis

Figure 1 depicts the clinical outcomes while on long-term vancomycin prophylaxis. One *C. difficile* relapse occurred during 220 person-months on vancomycin, for a breakthrough incidence of 4.5 per 1000 person-months. During the 9th week of long-term vancomycin prophylaxis, Patient 1, a 61-year-old male with three prior CDI episodes and no previous FMT treatment, developed acute diarrhea which prompted an increase in dosage to 250 mg four times a day for 2 weeks. Vancomycin was followed by 3 FMTs by rectal enema, and no further relapse was observed. Patient 1 illustrates the potential use of long-term vancomycin as a bridge to FMT when FMT is unavailable or if patients are currently not candidates for FMT.

In follow-up of 13 instances where vancomycin was discontinued, 4 (31%) relapses were observed within 8 weeks of discontinuation. All patients who relapsed after discontinuation of prolonged vancomycin were promptly retreated with a 14-day course of oral vancomycin four times a day, followed by a 2 week taper down to once daily oral vancomycin. The median time to relapse was 3.5 weeks (range, 2–6 weeks). Patient 5 is a 52-year-old female who had previously experienced 5 CDI episodes; she received 9 months of vancomycin, and relapsed 6 weeks after discontinuation. She was retreated with vancomycin four times daily, and then subsequently tapered back to daily vancomycin for life without further relapse. Patient 12 is a 74-year-old male who had experienced 5 CDI episodes before starting long-term vancomycin. Patient 12 relapsed 4 weeks after discontinuing 8 months of vancomycin, and relapsed again 3 weeks after discontinuing another 3.5 month course of vancomycin. He has since completed 3 months of long-term vancomycin prophylaxis without further relapse. Patient 18 is a 91-year-old female who had experienced 6 CDI episodes; she relapsed 2 weeks after completing 4.5 months of vancomycin and has been tapered back after acute treatment to once daily long-term vancomycin. In addition to the 4 relapses, 1 delayed episode of CDI was observed. Patient 7, a 68-year-old male with 6 prior episodes of CDI, experienced a CDI recurrence 5 months after completing 9 months of vancomycin; he is presently on life-long daily vancomycin prophylaxis. Given the prolonged time to a repeat CDI episode, Patient 7 likely represented re-infection rather than relapse. Four patients died during follow-up, but none of the deaths were ascribed to CDI. Three deaths were attributed to end-stage renal disease, and one to sepsis.

## Discussion

Recent guidelines suggest that there are insufficient data at this time to recommend an extension of antimicrobial treatment, beyond a typical course, for recurrent CDI [6]. This case series illuminates the potential for the use of long-term oral vancomycin for prophylaxis of recurrent or relapsing CDI. As this was a heavily pretreated elderly population, however, death was a competing outcome which could have led to an overstatement of the success rate. Moreover, our study is limited in size and in the non-systematic decision to start or discontinue prophylaxis. Further studies are needed to determine the optimum duration of prophylaxis, and to determine risk factors for relapse after discontinuing prophylaxis.

The indications for long-term oral vancomycin prophylaxis were decided clinically by one of three experienced infectious diseases physicians, and clinical judgment guided discontinuation of prophylaxis. It is possible that ongoing diarrhea may have represented *C. difficile* colonization or other diarrheal illnesses as no toxin testing was done while patients were on prolonged vancomycin. However, prior to the taper down to once daily vancomycin, all patients had persistent diarrhea and a positive test for *C. difficile* toxin gene which, based on the clinical judgement of three experienced infectious disease physicians, represented CDI-associated diarrhea. Overall, there was demonstrated safety at the 125 mg once daily dosage, as no allergies, adverse events, or instances of vancomycin-resistant enterococci were observed while patients were on long-term vancomycin.

## Conclusions

We conclude that prolonged vancomycin prophylaxis at a dose of 125 mg orally daily is an effective and well-tolerated option for secondary prevention of relapsing *C. difficile* infection, and may be considered in those without access to FMT, or who relapse or fail FMT.

## Abbreviations

*C. difficile*: *Clostridium difficile*
CDI: *Clostridium difficile* infection
FMT: Fecal microbiota transplantation
